# A Hybrid Methacrylate-Sodium Carboxymethylcellulose Interpolyelectrolyte Complex: Rheometry and *in Silico* Disposition for Controlled Drug Release

**DOI:** 10.3390/ma6104284

**Published:** 2013-09-26

**Authors:** Ndidi Chinyelu Ngwuluka, Yahya Essop Choonara, Pradeep Kumar, Girish Modi, Lisa Claire du Toit, Viness Pillay

**Affiliations:** 1 Faculty of Health Sciences, Department of Pharmacy and Pharmacology, University of the Witwatersrand, 7 York Road, Parktown, Johannesburg 2193, South Africa; E-Mails: ndidi.ngwuluka@students.wits.ac.za (N.C.N.); yahya.choonara@wits.ac.za (Y.E.C.); pradeep.kumar@wits.ac.za (P.K.); lisa.dutoit@wits.ac.za (L.C.D.T.); 2 Faculty of Health Sciences, Division of Neurosciences, Department of Neurology, University of the Witwatersrand, 7 York Road, Parktown, Johannesburg 2193, South Africa; E-Mail: gmodicns@mweb.co.za

**Keywords:** interpolyelectrolyte complex, eudragit, carboxymethylcellulose, rheology, hydrogel, molecular mechanics

## Abstract

The rheological behavioral changes that occurred during the synthesis of an interpolyelectrolyte complex (IPEC) of methacrylate copolymer and sodium carboxymethylcellulose were assessed. These changes were compared with the rheological behavior of the individual polymers employing basic viscosity, yield stress, stress sweep, frequency sweep, temperature ramp as well as creep and recovery testing. The rheological studies demonstrated that the end-product of the complexation of low viscous methacrylate copolymer and entangled solution of sodium carboxymethylcellulose generated a polymer, which exhibited a solid-like behavior with a three-dimensional network. Additionally, the rheological profile of the sodium carboxymethylcellulose and methacrylate copolymer with respect to the effect of various concentrations of acetic acid on the synthesis of the IPEC was elucidated using molecular mechanics energy relationships (MMER) by exploring the spatial disposition of carboxymethylcellulose and methacrylate copolymer with respect to each other and acetic acid. The computational results corroborated well with the experimental *in vitro* drug release data. Results have shown that the IPEC may be suitable polymeric material for achieving controlled zero-order drug delivery.

## 1. Introduction

Science and technology has resulted in various nanostructured materials to be rapidly developed, however the contributions of polymer-based materials still have a crucial role, especially rheological characterization of polymer blends or modified polymers. Rheological characterization of polymer blends or modified polymers are undertaken to assess the influence of the components as well as the interactions that may have occurred. While the size, shape, concentration and mode of particle size distribution influence the rheological properties of the blend, the interactions within the system influence the properties of the continuous medium [1]. Rheology is frequently employed to characterize pharmaceutical formulations especially emulsions, ointments, colloids and gels. Furthermore, the rheological properties of a polymer determines its suitability and application in pharmaceutical industry in terms of dosage forms, route of administration, site of action and the desired rate of release. Rheology is a useful tool, which provides indirect information on the structure and consistency, which refers to yield value, viscosity and loss factor; in turn, this influences sensory attributes and drug release [2]. Generally, rheological behaviors exhibited by polymer depend on the inter-chain interactions, crosslinking and entanglements; and relate to swelling, shrinking, erosion, degradation and diffusion of drugs [3,4,5,6,7,8]. For polyelectrolyte such as sodium carboxymethylcellulose (NaCMC), the conformation and rheological behavior exhibited is influenced by its ionization grade, which depends on the pH and ionic strength [4].

An interpolyelectrolyte complex (IPEC) was formed by the blending of a cationic polyelectrolyte and an anionic polyelectrolyte at a stoichiometric ratio. IPECs are employed as modified polymers in drug delivery, actuators in biotechnology and flocculants in various environmental and industrial processes [9]. NaCMC is a soluble semi-synthetic derivative of cellulose. Rheological characterization of NaCMC has been undertaken by several researchers [5,10,11,12,13,14,15]. It is known to exhibit a marked shear thinning flow pattern, visco-elastic properties and behaves as an entanglement polymer [16]. It is employed as viscosity-enhancing agent in food, cosmetics and pharmaceutical industries. Methacrylate copolymer (Eudragit^®^ E100) is a cationic polymer with low viscosity [17] that is soluble in acid medium while it swells in higher pH.

This study was undertaken to elucidate the rheological changes that occur during the synthesis of the IPEC of methacrylate copolymer and NaCMC. The comparative rheological studies undertaken between the individual polymers and the IPEC included basic viscosity, a yield stress test, oscillatory stress sweep, oscillatory frequency sweep, and temperature ramp, as well as creep and recovery testing. The *in silico* disposition data, *in vitro* drug release studies and the rheological properties of the IPEC demonstrated the potential of the polymeric material to be employed as a matrix for controlled zero-order drug release.

## 2. Results and Discussion

As the normality of acetic acid increased, the solubility of methacrylate copolymer increased. Furthermore, as methacrylate copolymer is added to NaCMC gel, the viscosity of the gel increased initially with visibly white strands running through the gel. However, on vigorous agitation, the viscosity decreased with time and thereafter increased towards the breaking point before it assumed its final viscosity. This was clearly observed where 0.1 N acetic acid was employed to dissolve methacrylate copolymer. The degree of reduction in viscosity is dependent on the degree of viscosity of NaCMC gel and the normality of acetic acid. As the normality of acetic acid increased from 0.1 N to 1.0 N, the viscosity of the end product decreased. At the stoichiometric ratio of 0.5 of methacrylate copolymer and 1.0 of NaCMC, there appeared to be complete polymer-polymer crosslinking or interaction as the end product was completely white (homogenous) and were like shreds which sedimented in low viscosity. It was envisaged the bonds within the NaCMC gel were broken faster at 0.8 and 1.0 N of acetic acid to generate IPEC with methacrylate copolymer in less than an hour unlike that of 0.1 N, which took about three hours. These visible observations during synthesis prompted the rheological studies of IPEC. The rheological properties of polymers employed for drug delivery can influence both *in vitro* and *in vivo* behaviors of a drug delivery system. The factors that determine the rheological outcome of the polymers include pH, temperature, polymer concentration, polymer modification, polymer combinations, ions and additives [18]. Hence, the need to study the rheological behavior of IPEC since some of the factors such as polymer concentration, modification, combination and ions were involved in its synthesis and may influence its drug release kinetics.

### 2.1. Basic Viscosity (η) of the Native Polymers and IPEC

**Figure 1:**
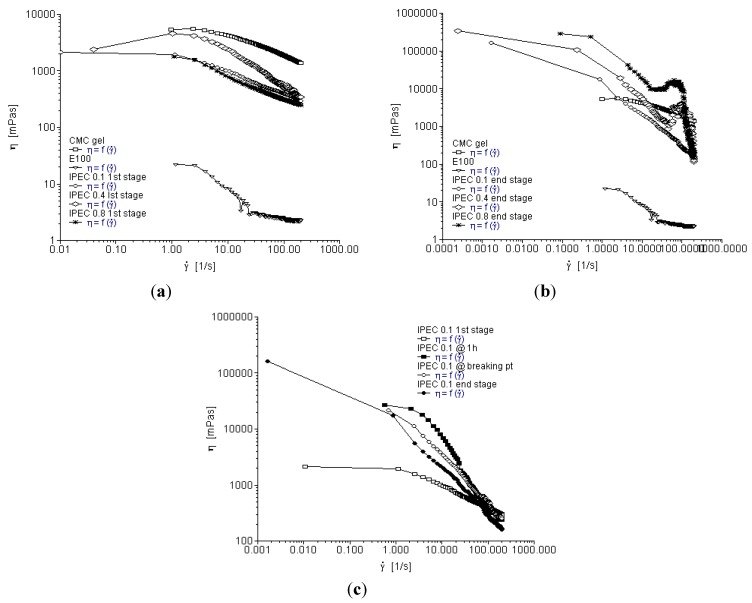
Viscosity as a function of shear rate for (**a**) the native polymers and interpolyelectrolyte complex (IPEC) at the first stage (sampling point I) of synthesis; (**b**) the native polymers and IPEC at the end stage (sampling point IV) of synthesis; and (**c**) IPEC at the 4 sampling points of the synthesis using 0.1 N acetic acid.

Methacrylate copolymer, NaCMC and IPEC generated from the interaction of methacrylate copolymer and NaCMC exhibited non-Newtonian flow pattern; for viscosity decreased as shear rate (γ) increased. Methacrylate copolymer is a polymer of low viscosity as can be seen from its rheogram depicting it as having the lowest viscosity while NaCMC is widely employed as a viscosity-enhancing agent. The IPEC like NaCMC exhibited shear thinning behavior and so could be said to be pseudoplastic which is also an indication that the interaction between the polymers did not alter shear thinning properties of the NaCMC (Figure 1a–c). Increasing shear rate (γ) forces the molecular chains to untangle which leads to molecular alignment making the molecules slip over each other with ease [19,20].

It is worthy to state that the same quantities of polymers were utilized for the rheological studies of the native polymers and IPEC. This means 1.68 g was used to determine the rheological properties of NaCMC and same quantity was used for the synthesis of IPEC and its rheological assessments. Initially at the onset of synthesis, the viscosity of the blends at the different normalities of acetic acid, 0.1, 0.4 and 0.8 N were less viscous than NaCMC (Figure 1a). However, at the end of synthesis, viscosity of IPEC increased compared to NaCMC but experienced more shear thinning than NaCMC with increased shear rate as depicted in Figure 1b. Decrease in viscosity with increased shear rate is attributed to the orientation or molecular deformation of the polymer network in the direction of the flow [21]. However, IPECs synthesized with 0.4 N and 0.8 N acetic acid at the end of synthesis behaved differently; for as shear rate increased, they exhibited shear thinning, shear thickening and then sharp shear thinning (Figure 1b). Perhaps, the order of untangling of molecular chains (molecular deformation) of the IPEC shreds may have been interrupted or slowed down as shear rate increased and then resumed as shear rate further increased. Gradients were calculated for each rheogram to quantify the impact of shear rate on the viscosities of IPEC at different sampling points and normality of acetic acid in comparison to NaCMC. The gradients at the end of synthesis were in the order: NaCMC (0.1757) < IPEC with 0.4 N acetic acid (0.3438) < IPEC with 0.1 N acetic acid (0.4353) < IPEC 0.8 N acetic acid (0.6554). Application of gradient confirmed that IPEC experienced more shear thinning than NaCMC for a higher gradient value indicated a steeper decrease in viscosity.

Figure 1c depicts viscosity *versus* shear rate for IPEC synthesized with 0.1 N acetic acid at the four stages of sampling during synthesis. The lowest viscosity of less than 5000 mPa·s was observed at the onset of synthesis and the highest viscosity of over 100,000 mPa·s was observed at the end of synthesis. The changes in viscosity can be attributed to the electrostatic interactions taking place within the two polymers [22]. The thickening or stiff behavior of IPEC at 1 h and breaking point could be due to the formation of entanglements of the polymer coils and increase in the intermolecular interactions [11]. In addition, shear thinning was more with IPEC 0.1 N at 1 h and breaking point than at the end stage of synthesis which was confirmed by gradient values (0.5318 at 1 h, 0.4435 at breaking point and 0.4353 at the end). For one hour and breaking point, rheograms were only obtained for IPEC synthesized using 0.1 N acetic acid (Figure 1c). The rate of synthesis is accelerated as the normality of acetic acid increased, so rheograms could not be obtained for 0.4 N and 0.8 N acetic acid at one hour and breaking point as the synthesis reached completion at one hour. Increase in normality increases the concentration of acetic acid, which in turn increases the rate of reaction/synthesis. Acetic acid stabilizes the ammonium cations of the methacrylate copolymer as well as reacting with sodium ions from NaCMC to produce sodium acetate that acted as a catalyst to increase the rate of reaction. In addition, as the concentration (normality) increased, the probabilities of particle collision increased thereby further increasing the rate of reaction. The effect of acetic acid was modeled and further elucidated later in this paper (Section 2.7.3).

The model that best describes the viscosity *versus* shear rate curve above is the cross model:

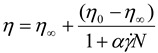
(1)
where *α* and *N* are constants while *γ* is the shear rate. The constant *N* was mostly 2/3 in a wide variety of materials tested while α is described as the characteristic shear rate at which the apparent viscosity of the system is the mean of the two limiting values *η*_0_ (viscosity at zero shear rate) and *η*_∞_ (viscosity at infinite shear rate) [23].

### 2.2. Yield Stress Analyses

**Figure 2:**
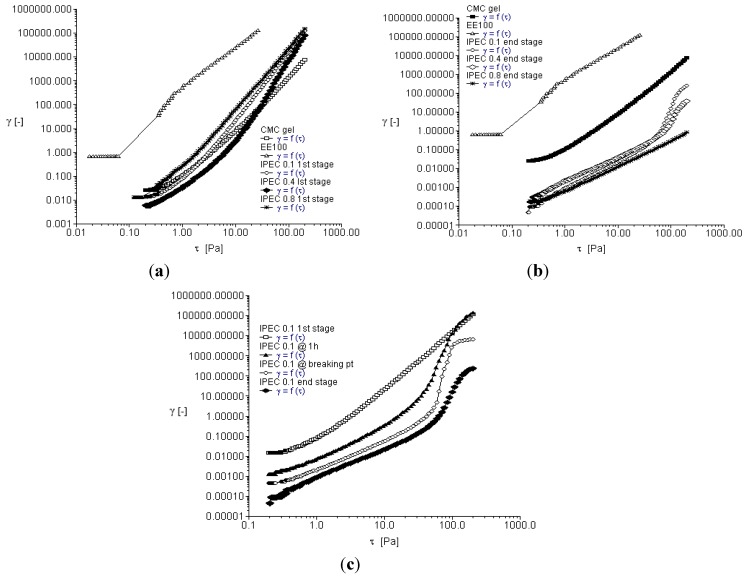
Yield stress rheogram for (**a**) the native polymers and IPEC at the first stage of synthesis; (**b**) the native polymers and IPEC at the end stage of synthesis; and (**c**) IPEC at the four sampling points of synthesis using 0.1 N acetic acid.

Yield stress is the point or stress above which a substance flows due to deformation of its internal structure. Below the yield value, a material is a solid exhibiting elasticity under small stresses and strains [24] and above the yield value the material behaves as a liquid with plastic viscosity [25]. Although Barnes and Walters argues there is no real yield stress as fluids that flow in high stresses can also flow in small stresses [25], the apparent yield stress is valuable. A material may generate apparent yield stress if studied in a shear stress *versus* shear rate region just above the large increase in viscosity [26]. Yield stress tests were undertaken to obtain the yield values of the native polymers and IPEC to provide insight on their yield strength as the IPEC would be employed in matrix formation. The shear (γ) *versus* shear stress (τ) rheograms was used to determine yield points for the native polymers and IPEC which are shown in Figure 2a–c while the apparent yield values calculated using the software RheoWin PC version 3 are given in Table 1.

The yield stress, which is an indication of internal strength of IPEC 0.1 N, increased from the initial stage of synthesis to its completion. In comparison to NaCMC, the yield value of IPEC 0.1 N (Table 1) is significantly higher. An increase in yield value represents a strengthening of the three-dimensional network structure of the polymer [18]. Crosslinking NaCMC with methacrylate copolymer enhanced its polymeric structure making it more elastic and thereby improving the mechanical strength of NaCMC. Therefore, it is envisaged that matrices formed with IPEC will have improved physicomechanical properties. It was observed that the yield values (resistance to flow) of IPEC synthesized with 0.4 N and 0.8 N acetic acid (at the end stage) were lower than that of IPEC synthesized with 0.1 N acetic acid. The IPEC synthesized with 0.1 N acetic acid, exhibiting a more solid-like behavior due to enhanced internal strength from the degree of molecular chain associations. Hence, the application of IPEC may determine the normality of acetic acid that will be employed for synthesis.

**Table 1:** Yield values of native polymers and IPEC at the sampling stages during synthesis.

Sample	Yield value (Pa)	Shear	Time (s)
NaCMC	0.2911	0.02802	31.47
E100	0.07212	0.9648	32.17
IPEC^1^ 0.1 N initial stage	0.2482	0.01442	31.22
IPEC 0.1 N @ 1h	0.2356	0.001318	30.98
IPEC 0.1 N breaking pt	41.42	0.9988	72.35
IPEC 0.1 N final stage	53.31	0.5160	83.77
IPEC 0.4 N initial stage	0.2904	0.006955	31.13
IPEC 0.4 N final stage	0.2311	0.0001735	30.67
IPEC 0.8 N initial stage	0.1923	0.01345	30.66
IPEC 0.8 N final stage	0.3329	0.0001819	31.03

^1^ denotes Complex of Eudragit E100 and sodium carboxymethylcellulose (NaCMC).

### 2.3. Oscillation Stress Sweep

**Figure 3:**
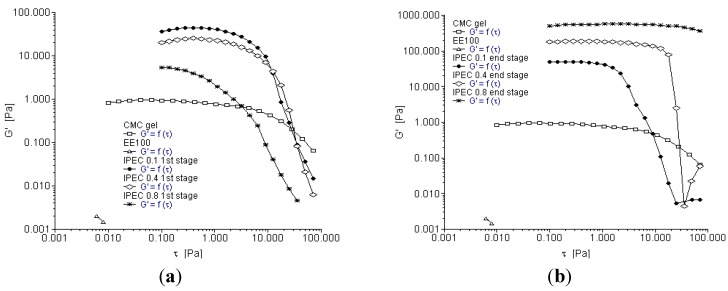

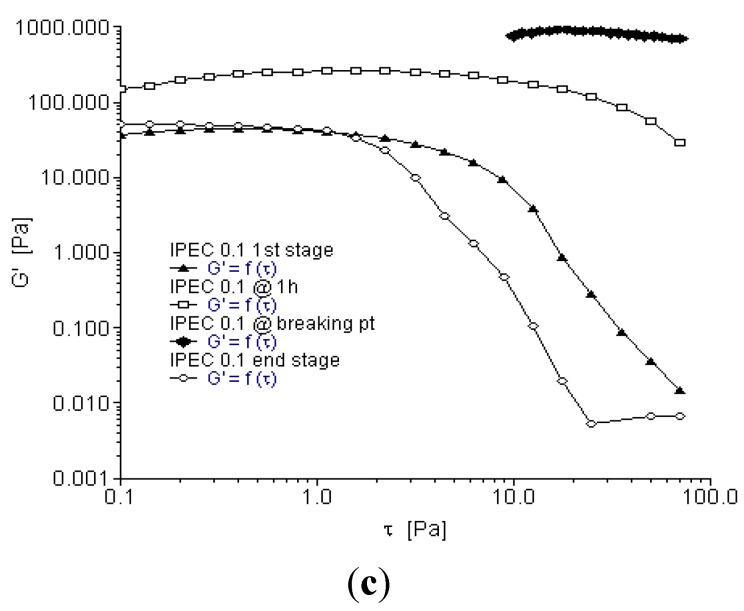
Elastic modulus as a function of shear stress for (**a**) the native polymers and IPEC at 1st stage of synthesis at frequency of 0.1 Hz; (**b**) the native polymers and IPEC at end stage of synthesis at frequency of 0.1 Hz; and (**c**) IPEC 0.1 N at the 4 sampling points of synthesis at frequency of 0.1 Hz.

Oscillation stress sweep is used to obtain the visco-elastic region of a material. The visco-elastic region will impact on the mechanical strength of IPEC, which will in turn influence the drug release kinetics when used as a drug carrier. The visco-elastic region is frequency dependent and so stress sweep was performed over three frequencies in this study (0.1, 1 and 10 Hz). The strength and subsequently the stability of a material correlate with its yield value. As the elastic modulus (*G*′) begins to decrease, the material undergoes structural deformation. The stress at which the material begins to exhibit non-linear behavior is the yield stress. The increase in elastic modulus also relates to the degree of intermolecular crosslinking within the polymer network [27]. Furthermore, degree of crosslinking correlates with the increase in yield stress and subsequently the extension of the visco-elastic region. Crosslinking increases the elastic modulus of a polymer thereby increasing its mechanical strength [28]. Storage or elastic modulus G′ represents the elastic property of the polymer while loss modulus *G*′′ represents the viscous properties. Figure 3a–c depict elastic modulus *G*′ as a function of shear stress at frequency 0.1 Hz. It is apparent methacrylate polymer lacked elasticity and hence showed no evidence of a visco-elastic region. On addition of methacrylate copolymer into NaCMC, elastic modulus of IPEC was increased (Figure 3a,b). However, compared to NaCMC, IPEC visco-elastic region is not as extended. As shear stress increased, the resistance to deformation decreased. As the synthesis proceeded for one hour, the elastic modulus of IPEC 0.1 N increased (Figure 3c) and continued increase was observed at breaking point with higher resistance to deformation. At the breaking point, there were large intermolecular forces enabling inter-chain crosslinking to occur between NaCMC and methacrylate copolymer, which in turn caused increased resistance to deformation and the polymer complex behaved as a solid. It is envisaged that significant increase in elastic modulus at breaking point could be due to shear induced transformation of intramolecular chain associations to intermolecular chain associations [29]. On completion of the associations, the elastic modulus was reduced and it is envisaged that this was due to little or no complexing ions left and on continued shear stress, the polymer succumbed to deformation. Contrary to the initial stage of synthesis, the end stage indicated that the elastic modulus increased as normality of acetic acid increased.

**Figure 4:**
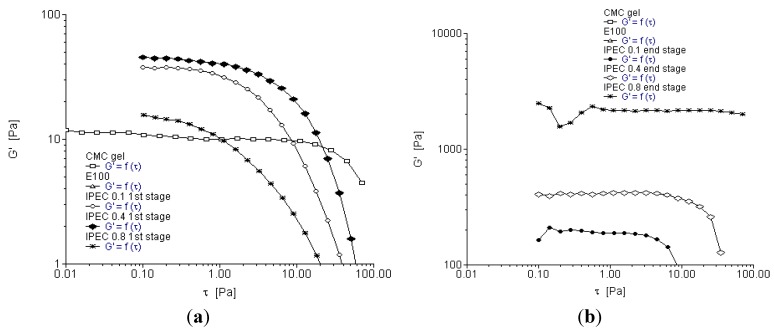

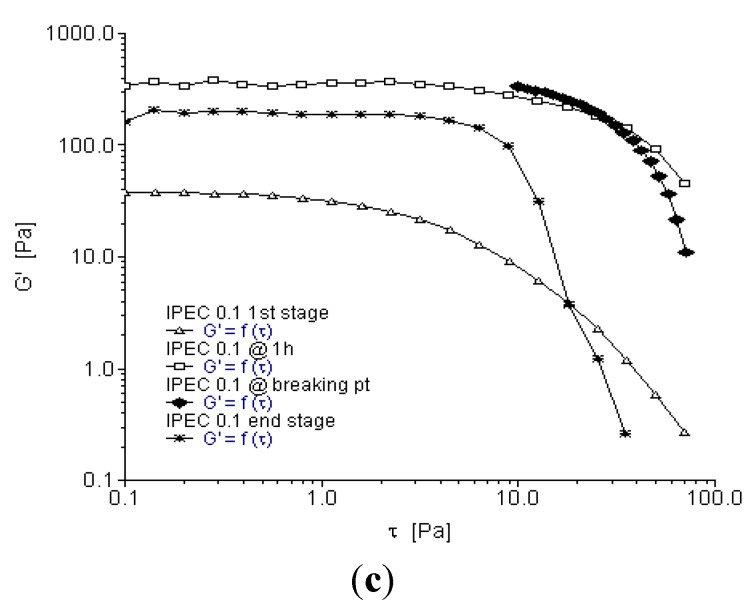
Elastic modulus as a function of shear stress for (**a**) the native polymers and IPEC at initial stage of synthesis at frequency of 1 Hz; (**b**) the native polymers and IPEC at end stage of synthesis at frequency of 1 Hz; and (**c**) IPEC 0.1 N at the 4 sampling points of synthesis at frequency of 1 Hz.

**Figure 5:**
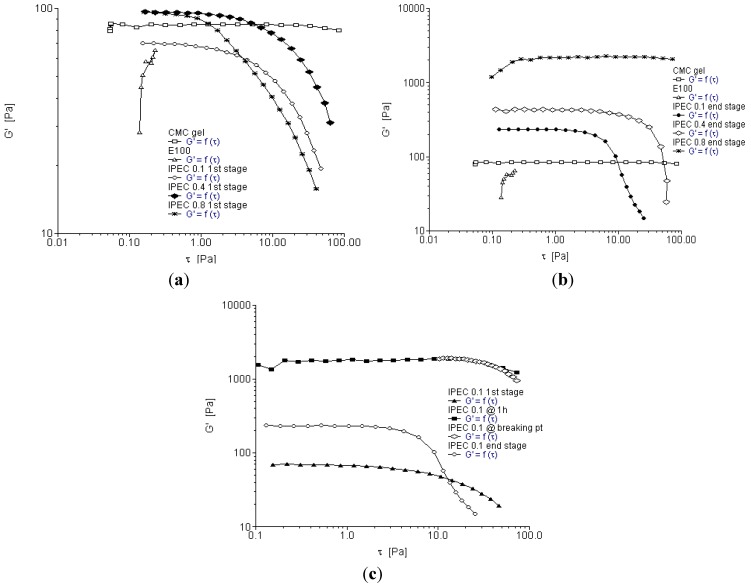
Elastic modulus as a function of shear stress for (**a**) the native polymers and IPEC at the first stage of synthesis at frequency of 10 Hz; (**b**) the native polymers and IPEC at end stage of synthesis at frequency of 10 Hz; and (**c**) IPEC 0.1 N at the 4 sampling points of synthesis at frequency of 10 Hz.

Figure 4a–c depict elastic modulus as a function of shear stress at frequency 1 Hz while Figure 5a–c depict elastic modulus as a function of shear stress at frequency 10 Hz. Generally, elastic modulus increased with increase in frequency. NaCMC’s elastic modulus increased from 1 Pa at frequency 0.1 Hz to a little above 10 Pa at frequency 1 Hz and almost 100 Pa at frequency 10 Hz while the final product of IPEC 0.8 N was well over 1000 Pa at frequency 1 Hz and 10 Hz. The visco-elastic region was also found to be extended for IPEC as frequency increased. At the initial stage of synthesis, it seemed NaCMC was more resistant to deformation; however, by one hour into synthesis, the increased strength of the internal structure of IPEC became evident indicating improved mechanical strength by crosslinking as IPEC required increased shear stress to deform and flow as a liquid. Furthermore, as normality of acetic acid employed increased, the shear stress required for deformation increased. From the visco-elastic regions the yield points were obtained and utilized in frequency sweep test for each sampling stage.

### 2.4. Oscillation Frequency Sweep

**Figure 6:**
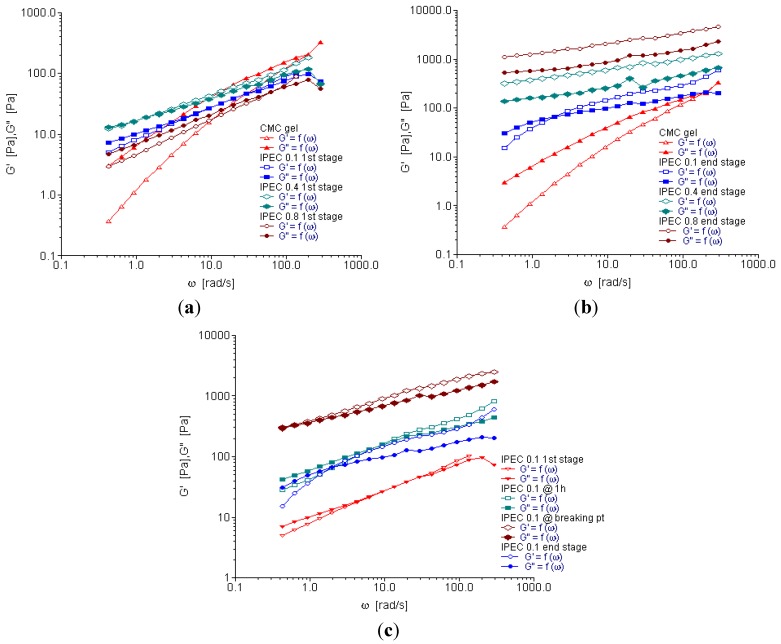
Elastic and viscous modulus as a function of angular frequency for (**a**) NaCMC and IPEC at 1st stage of synthesis; (**b**) NaCMC and IPEC at end stage of synthesis; and (**c**) IPEC 0.1 N at the 4 sampling points of synthesis.

Frequency sweep is used to characterize materials in terms of their various structures. It can be used to determine if a material is a three-dimensional network, an entangled solution or a particle solution: in order words gel, paste or liquid. The rheograms depicted in Figure 6a–c showed that elastic and loss moduli increased with increasing frequency (angular frequency—ω). However, for NaCMC, loss modulus was higher than elastic modulus until the frequency increased to about 500 rad/s; then a crossover took place and elastic modulus became slightly higher (Figure 6a,b). This is an indication that for most part of the test, NaCMC behaved as a viscous liquid. On the contrary, IPECs with the different normalities of acetic acid were elastic as the elastic moduli were higher than the loss moduli. Generally, IPEC exhibited a solid-like behavior. However, IPEC 0.1 N at one hour into synthesis exhibited viscous behavior at low frequency, then there was a crossover and it became elastic as the frequency increased behaving as an entangled solution (Figure 6c). The final product of IPEC 0.1 N behaved similarly. The elastic and loss moduli of IPEC 0.1 N at breaking point rheogram merged at a lower frequency (below 10 rad/s); however as the frequency began to increase towards 10 rad/s, elastic modulus was higher than loss modulus. At the beginning of the test, it showed a visco-elastic behavior and as the test continued and frequency increased it was elastic indicating and confirming its high resistance to deformation and subsequent high yield value. The elastic and loss moduli for IPEC 0.4 and IPEC 0.8 N were basically parallel; with elastic moduli higher than loss moduli which indicates that they behaved as three-dimensional network (Figure 6b). At lower normality of acetic acid, entangled solution was obtained while higher normality of acetic acid generated a gel—an improvement on NaCMC, which behaved as a viscous liquid. Hence, IPEC has improved structural ability, which suggests it may be used for formulation of matrix tablets and possibly hydrogel for controlled release. The gel elasticity is articulated on the assumption that the reference mean square end-to-end length distance “*r*” of a network strand corresponds to the conformation of the polymer chain would appropriate if it were in a solution at the same concentration as the gel [30]. Hence, elastic modulus is:

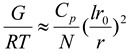
(2)
where *R* is the gas constant, *N* is the number of monomers between two crosslinks, *r*_0_ is the mean square end-to-end length of the polymer chain at the preparation state and *l* is a linear expansion coefficient [30].

Furthermore, during the crosslinking of a polymer, the gel point is determined by Winter-Chambon criteria. However, gel point can also be determined by plotting of *G*′*G*′′ at various frequencies and gel point can be represented by a simple power law; the Kramers–Kroenig relationship:

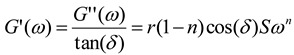
(3)
where, phase angle *δ* = *n*π*/*2, or tan*δ* (=tan(*n*π*/*2)) is independent of frequencies, and *S* is the gel strength (Pa·s*^n^*) [31].

One of the models used to predict a polymer’s response to various loading conditions is Maxwell’s model. This model infers that stress decays exponentially with time [32]. During the oscillatory test of a Maxwell model whereby *G*′ and *G*′′ are in combination, the response gives a more complex and pronounced behavior and is represented mathematically [26]:

(4) $$G\prime \prime \text{=}\frac{n\omega }{1+{\left(\omega \tau \right)}^{2}}\;and\;G\prime =\frac{{G\left(\omega \tau \right)}^{2}}{1+{\left(\omega \tau \right)}^{2}}$$

where relaxation time *τ* = η/*G*·*G*′′ is larger than *G*′ at low frequencies leading to the dominance of liquid-like behavior. However, at higher frequencies, *G*′ increases becoming larger than *G*′′ and hence solid-like behavior dominates [26].

### 2.5. Creep and Recovery Test

**Figure 7:**
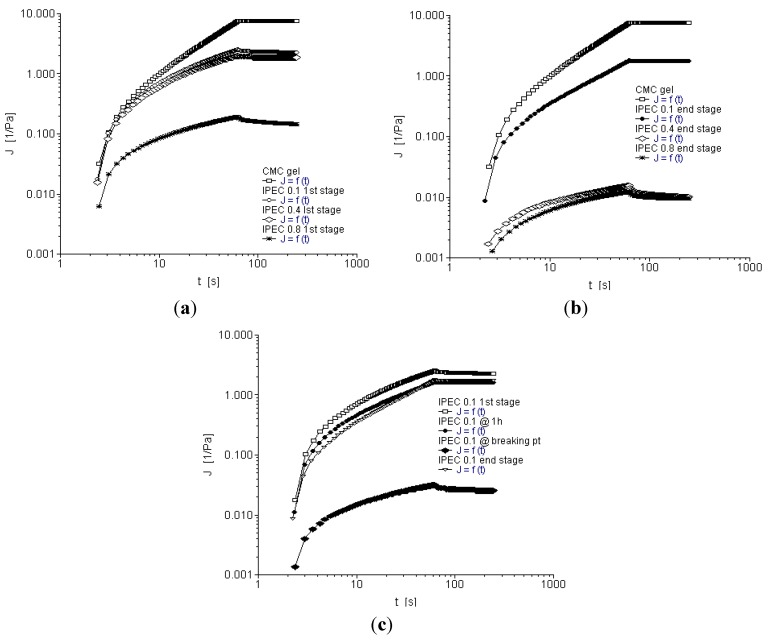
Creep recovery rheogram for (**a**) NaCMC and IPEC at 1st stage of synthesis; (**b**) NaCMC and IPEC at end stage of synthesis; and (**c**) IPEC 0.1 N at the 4 sampling points of synthesis.

When stress is applied, a material is easily deformed if the material has higher compliance (J) [4,12]. Creep recovery test helps to assess the elasticity of a material—when stress applied is removed, the materials after deformation may recoil and attempt to restore back to its original shape. Furthermore, the level of deformation is dependent on the period of stress, the amount of stress/strain applied and temperature. High amount of creep strain during creep test will result in higher residual strain in recovery [33]. A slow recovery indicates that the material stores residual stress [12]. The rheograms show elastic deformation as well as elastic recovery within the applied stress. The rheograms are non-linear which is indicative of elastic recovery of NaCMC and IPEC as well as viscous behavior. From Figure 7a,b, it could be observed that within the stress applied, the native polymer, NaCMC and IPEC at the different normalities of acetic acid showed elastic deformation and when the stress was removed, they were able to recover. However, IPEC 0.1 N at breaking point (Figure 7c) did not fully recover like the others showing the polymer is not as stable at that stage of synthesis. The rheograms of IPEC had lower compliance than NaCMC; an indication that it is not as easily deformed as NaCMC, which suggests and confirms that polymer-polymer crosslinking may have enhanced its elasticity and mechanical strength. Comparison among IPECs showed that as normality of acetic acid increased, the compliance decreased; however, the lower normality (0.1 N) has a more superior recovery which may suggest that the lower normality (0.1 N) has slightly more ability to recoil and restore to original shape faster than the higher normalities (Figure 7b).

### 2.6. Temperature Ramp

**Figure 8:**
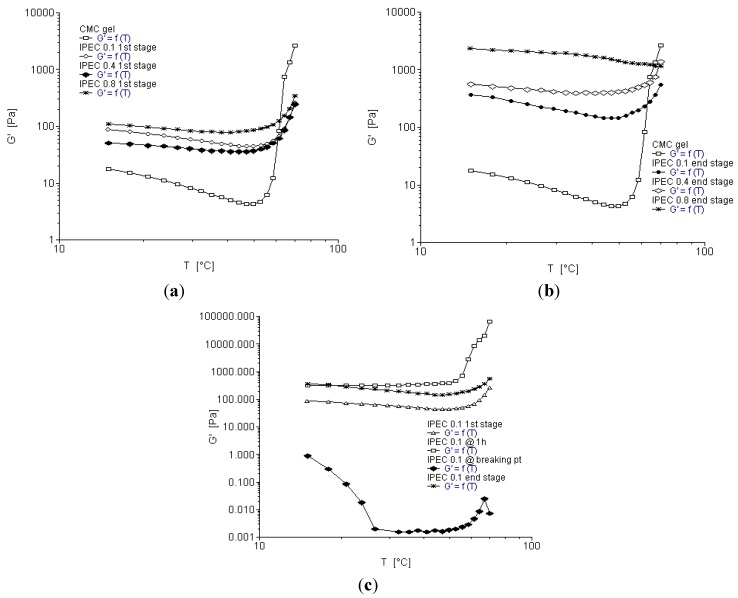
Elastic modulus as a function of temperature for (**a**) NaCMC and IPEC at the first stage of synthesis; (**b**) NaCMC and IPEC at the end stage of synthesis; and (**c**) IPEC 0.1 N at the four sampling points of synthesis.

Temperature ramp is used to observe the change in the properties of a polymer due to temperature. The stress or strain applied is within the visco-elastic region. Figure 8a–c depict rheograms of elastic modulus as a function of temperature. The elastic modulus of NaCMC decreased slightly with increase in temperature and then began to increase after 50 °C. Initial increase in temperature increased slightly the fluidity of NaCMC, which is confirmed by several studies [34,35,36,37]. Elastic moduli for IPEC 0.4 N, IPEC 0.8 N and IPEC 0.1 N at one hour into synthesis appeared independent of temperature until about 60 °C after which a slight increase of elastic modulus is observed. The elastic moduli of IPEC 0.1 N at breaking point was clearly decreased by temperature. Attempt to increase temperature at breaking point may affect the intramolecular as well as intermolecular interactions causing them to separate instead of interact thereby increasing the fluidity of the polymer complex. However, the elastic modulus of the polymer complex at the end of synthesis was not affected significantly by temperature (Figure 8b). The slight effect of temperature on IPEC at the end of synthesis suggests it is not thermo-sensitive.

### 2.7. Molecular Mechanics Assisted Model Building and Energy Refinements

A molecular mechanics conformational searching procedure was employed to acquire the data employed in the statistical mechanics analysis, and to obtain differential binding energies of a Polak–Ribiere algorithm and to potentially permit application to polymer composite assemblies. MM+ (force field) is a HyperChem™ modification and extension of Norman Allinger’s Molecular Mechanics program MM2 [38] whereas AMBER, is a package of computer programs for applying molecular mechanics, normal mode analysis, molecular dynamics and free energy calculations to simulate the structural and energetic properties of molecules [39].

#### 2.7.1. MMER Analysis

Molecular mechanics energy relationship (MMER), a method for analytico-mathematical representation of potential energy surfaces, was used to provide information about the contributions of valence terms, non-covalent Coulombic terms, and non-covalent van der Waals interactions for solute partitioning from the bulk phase. The MMER model for potential energy factor in various molecular complexes can be written as:

*E*_molecule/complex_ = *V*_∑_ = *V*_b_ + *V*_θ_ + *V*_φ_ + *V*_ij_ + *V*_hb_ + *V*_el_ (5)

where, *V*_∑_ is related to total steric energy for an optimized structure, *V*_b_ corresponds to bond stretching contributions (reference values were assigned to all of a structure's bond lengths), *V*_θ_ denotes bond angle contributions (reference values were assigned to all of a structure's bond angles), *V*_φ_ represents torsional contribution arising from deviations from optimum dihedral angles, *V*_ij_ incorporates van der Waals interactions due to non-bonded inter-atomic distances, *V*_hb_ symbolizes hydrogen-bond energy function and *V*_el_ stands for electrostatic energy.

In addition, the total potential energy deviation, Δ*E*_Total_, was calculated as the difference between the total potential energy of the complex system and the sum of the potential energies of isolated individual molecules, as follows:

Δ*E*_Total(A/B)_ = *E*_Total(A/B)_ − (*E*_Total(A)_ + *E*_Total(B)_) (6)

The molecular stability can then be estimated by comparing the total potential energies of the isolated and complexed systems. If the total potential energy of IPEC is smaller than the sum of the potential energies of isolated individual molecules (NaCMC and E100) in the same conformation, the IPEC is more stable and its formation is favored [40]. The monomer length for the polymer chain depicting molecular structures of the polymers were determined on the basis of equivalent grid surface area covered by the polymers so that the inherent stereo-electronic factors at the interaction site can be perfectly optimized. The set of low-energy conformers that were in equilibrium with each other was identified and portrayed as lowest energy conformational model.

In the present SLAS study, a set of three compounds were used to build the energy models for the polyelectrolytic systems. The synthesis and stability properties of the IPEC entities that vary in rate of formation were studied using this polymer/solvent system (IPEC/acetic acid). The global energy relationships for the various complexes derived after assisted model building and energy refinements are as follows:

*E*_CMC_ = −9.605*V_∑_* = 3.024*V_b_* + 19.746*V_θ_* + 32.574*V_φ_* + 8.459*V_ij_* − 1.759*V_hb_* − 71.649*V_el_* (7)

*E*_E100_ = 100.577*V_∑_* = 10.018*V_b_* + 45.058*V_θ_* + 13.454*V_φ_* + 32.052*V_ij_* − 0.006*V_hb_* (8)

*E*_IPEC-I_ = 90.972*V_∑_=* 13.043*V_b_* + 64.804*V_θ_* + 46.028*V_φ_* + 40.512*V_ij_* − 1.767*V_hb_* − 71.649*V_el_* (9)

*E*_IPEC-II_ = 72.9*V_∑_* = 13.13*V_b_* + 65.816*V_θ_* + 46.161*V_φ_* + 21.409*V_ij_* − 2.509*V_hb_* − 71.108*V_el_* (10)

*E*_IPEC-III_ = 69.543*V_∑_ =* 12.607*V_b_ +* 66.001*V_θ_+* 43.271*V_φ_* + 19.977*V_ij_* − 4.265*V_hb_* − 68.049*V_el_* (11)

*E*_IPEC-IV_ = 66.219*V_∑_=* 12.184*V_b_ +* 63.708*V_θ_+* 45.470*V_φ_* + 14.936*V_ij_* − 3.919*V_hb_* − 66.161*V_el_* (12)

*E*_IPEC-Sol_ = −5426.791*V_∑_* = 61.652*V_b_* + 115.959*V_θ_* + 55.433*V_φ_* + 61.924*V_ij_* − 18.994*V_hb_* − 5702.76*V_el_* (13)

*E*_IPEC-AcA2_ = −5501.659*V_∑_* = 60.073*V_b_* + 119.566*V_θ_* + 56.049*V_φ_* + 61.745*V_ij_* − 15.457*V_hb_* − 5791.63*V_el_* (14)

*E*_IPEC-AcA4_ = −5520.468*V_∑_* = 60.102*V_b_* + 119.615*V_θ_* + 56.075*V_φ_* + 50.928*V_ij_* − 19.303*V_hb_* − 5787.89*V_el_* (15)

*E*_IPEC-AcA8_ = −5539.437*V_∑_* = 59.302*V_b_* + 118.963*V_θ_* + 58.314*V_φ_* + 34.261*V_ij_* − 25.67*V_hb_* − 5784.61*V_el_* (16)

#### 2.7.2. Elucidation of Step-by-Step Synthetic Profile of IPEC

A novel technique was employed for modeling the sampling points of the synthesis of an IPEC using an Intermittent Snapshot Modeling Approach (ISMA). The snapshot was taken just after a major change in molecular conformation during energy minimization and energy values calculated by pausing the minimization process. The various energy equations generated using MMER for the step-by-step elucidation are represented by Equations 7–12 where *E*_IPEC-I_, *E*_IPEC-II_, *E*_IPEC-III_, and *E*_IPEC-IV_ correspond approximately to first (30 s after methacrylate copolymer solution was added into NaCMC solution), second (one hour later), third (at breaking point), and fourth (end of synthesis) stages, respectively.

**Figure 9:**
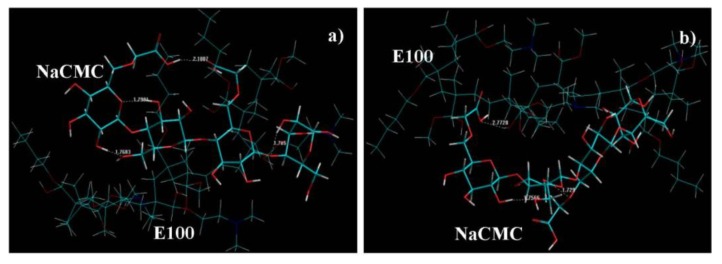

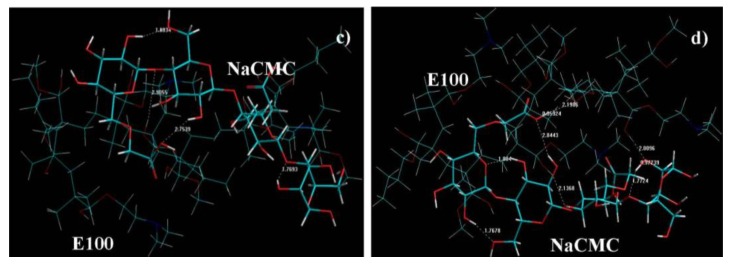
Visualization of geometrical preferences of the NaCMC-E100 (IPEC) complex: (**a**) first stage: initial mixing of two polymers; (**b**) second stage: after 1 h; (**c**) third stage: breaking point; and (**d**) fourth stage: final product after Molecular Mechanics simulations. Color codes: C (cyan), O (red), N (blue) and H (white).

As evident from the energy Equations (7)–(12) and Table 2, all the stages were accompanied by a significant decrease in total steric energy as compared to the respective previous stage with E_IPEC-IV_ having the least energy. No particular trend was observed during the formation of different stages as the component energy minimizations varied around the spatial arrangements of the reacting functional groups. The bond energy and van der Waals forces underwent minimization as the reaction progressed. Alternatively, electrostatic interactions displayed upward energetic trajectory. The difference in the visco-elastic behavior at all the stages can be predicted from a close look at the geometrical conformations generated after Molecular Mechanics simulations. It is proposed that change in viscosity may be due to breaking and forming of intramolecular (NaCMC) and intermolecular (NaCMC-E100) bonds and interactions as shown in Figure 9 and as discussed below:

1. At the initial stage (where the E100 was added to NaCMC), numerous intramolecular bonds in NaCMC were observed with no intermolecular bonding between E100-NaCMC (Figure 9a). This is confirmed by the initial formation of a gel phase by NaCMC in the reaction medium observed at the onset of synthesis.
2. At 1h into synthesis, it was observed that NaCMC intramolecular bonds reduced in number and bond-length also decreased along with formation of an intermolecular bond with E100 depicting the first evidence of formation of an IPEC. This is corroborated experimentally by the increase in elastic modulus at one hour into synthesis (Figure 3c, Figure 4c and Figure 5c). The formation of intermolecular bonding enable crosslinking between E100 and NaCMC leading to increased elastic modulus.
3. At breaking point, again the intramolecular bond of NaCMC started rebuilding with a slight increase in the length of the intermolecular bonds. This supports observations during synthesis and rheological studies. A thickening of the reaction medium, increase in elastic modulus and shear stress occurred at this stage which was generated by the entanglements of NaCMC coils due to rebuilding of intramolecular bonds and then gradual increase in intermolecular bonds between E100 and NaCMC.
4. The formation of final product displayed a homogenous IPEC, which was accompanied with the formation of numerous intra- and inter-molecular bonds depicting the formation of a well interconnected polymeric matrix. Additionally, the bond length also seemed to be playing a part as the average bond length decreased to <2Å. The formation of numerous bonds yielded a polymeric matrix with increased resistance to flow as observed with increased yield values in comparison to the individual polymers.

**Table 2:** Computational differential energy attributes calculated for the simulated IPEC system in a molecular mechanics’ force field setup performed using HyperChem™ 8.0.8 (Hypercube Inc., Gainesville, FL, USA).

Name	Δ*E*_Total_^1^	Δ*E*_Bond_	Δ*E*_Angle_	Δ*E*_Dihedral_	Δ*E*_Vdw_	Δ*E*_H-bond_	Δ*E*_Elec_
E_IPEC-II_	−18.072	0.087	1.012	0.133	−19.103	−0.742	0.541
E_IPEC-III_	−21.429	−0.436	1.197	−2.757	−20.535	−2.498	3.600
E_IPEC-IV_	−24.753	−0.859	−1.096	−0.558	−25.576	−2.152	5.488

^1^ Δ*E* calculated with in comparison to the energy values of the first stage.

#### 2.7.3. Effect of Normality of Acetic Acid on the Reactional Profile of IPEC

**Figure 10:**
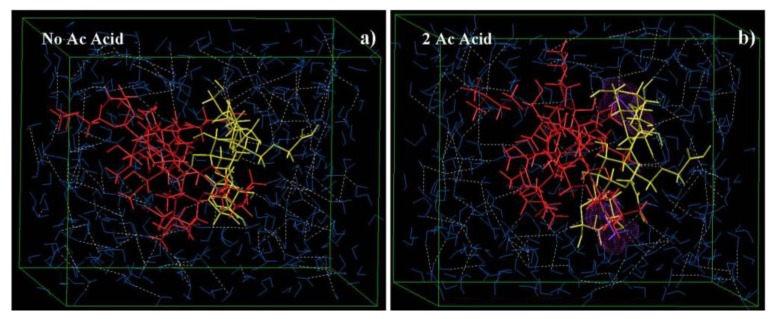

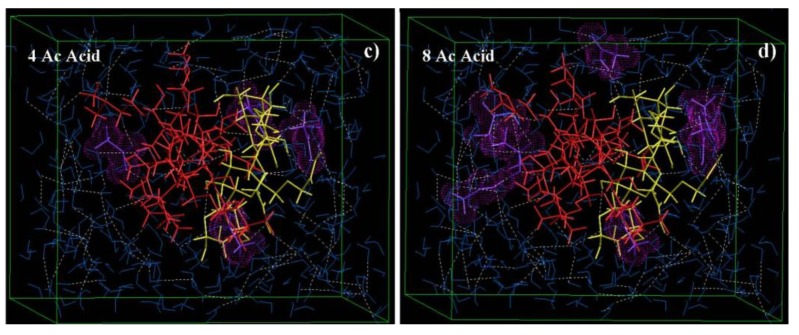
Visualization of geometrical preferences of IPEC molecule presence of (**a**) no acetic acid molecules; (**b**) 2 acetic acid molecules; (**c**) 4 acetic acid molecules; and (**d**) 8 acetic acid molecules after molecular simulation in a solvated system consisting of water molecules. Color codes for molecules: NaCMC (yellow), E100 (red), Acetic acid (purple), and water (blue).

Experimentally, an increase in normality of acetic acid increased the rate of synthesis. An attempt was made to explicate the mechanisms by which this is achieved using Molecular Mechanics simulations in a solvated system. As evident from Equations (13)–(16), we observed a specific trend during the simulations: higher the normality of the acid used, higher was the stability obtained (total energy decreases). This means that the reaction will go faster towards completion with an increase in normality. It was further confirmed by the fact that the rate of synthesis was accelerated as the normality of acetic acid increased and hence rheograms could not be obtained for 0.4 N and 0.8 N acetic acid at one hour and breaking point since the synthesis reached completion at one hour. Furthermore, interestingly, the end product remained almost the same as the bonding interaction remained almost the same as depicted by the bond, angle and dihedral energies [Figure 10; Equations (13)–(16)]. However, as the normality increased the electrostatic forces also increased giving rise to a trend of increased repulsion among the constituent polymer molecules. This doesn’t mean that the IPEC would break with time as it seems to remain intact because of the highly stabilized van der Waals forces and H-bonding. The increase in repulsion among the constituent polymer molecules as normality increased may explicate the decreased yield values observed as normality of acetic acid increased.

### 2.8. Drug Release Profiles

**Figure 11:**
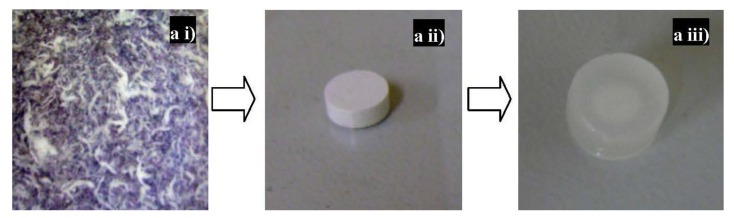

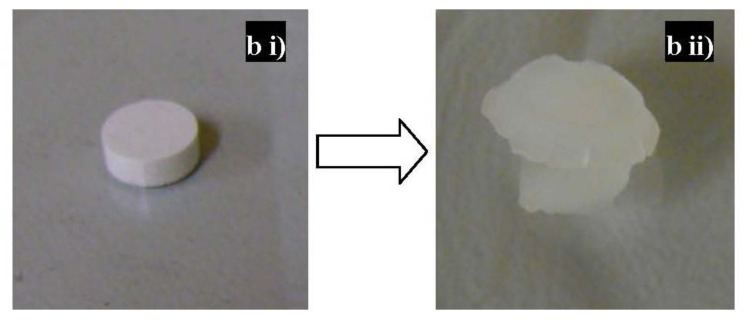
Digital images of (**a**) transition of IPEC from (**i**) synthesis to (**ii**) compression into tablet matrix and (**iii**) *in vitro* drug release retaining its three dimensional network; (**b**) NaCMC after (**i**) compression into tablet matrix and (**ii**) *in vitro* drug release losing its three dimensional network.

**Figure 12:**
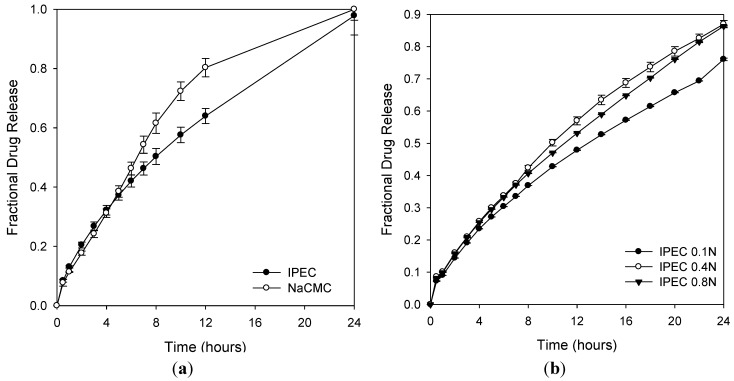
Comparative *in vitro* drug release profiles of (**a**) NaCMC and IPEC; and (**b**) IPEC at different normalities of acetic acid: 0.1 N, 0.4 N and 0.8 N.

Swelling is one of the mechanisms of drug release. Solvent penetrates the tablet matrix, it swells and the drug dissolves and diffuses out through the pores. The rate of diffusion is dependent on the degree of swelling thereby influencing the rate of drug release from the matrices. The degrees of swelling for NaCMC and IPEC were 384% and 465%, respectively. This is indicative that the interaction of NaCMC with methacrylate copolymer may have improved the ability of NaCMC to be retained in the stomach due to excessive swelling exhibited. Hence, its gastroretentive potential may have been enhanced by the complexation. Furthermore, NaCMC lost its three-dimensional network while IPEC did not (Figure 11). NaCMC began to lose its three-dimensional network visibly form the 3rd hour. IPEC retaining its three-dimensional network indicates that it has more superior physicomechanical strength than NaCMC. Initially, the rate of release from NaCMC matrices was slower than that of IPEC; however, as time progressed, the rate of release increased such that the NaCMC matrices released averagely 80% of levodopa by the 12th hour while IPEC matrices released 64% (Figure 12). IPEC exhibited a more controlled release of levodopa over time at a constant rate. Hence IPEC shows promise as a superior material with improved drug delivery properties for controlled (extended or modified) release that can achieve zero order kinetics. This preliminary drug release studies corroborates the rheological analyses, which suggested that IPEC behaved as a three-dimensional network with improved structural ability suitable for formulations of tablet matrices.

## 3. Experimental Section

### 3.1. Materials

Methacrylate copolymer based on dimethylaminoethyl methacrylate, butyl methacrylate and methyl methacrylate with a ratio of 2:1:1 was purchased. It was soluble in organic solvents such as ethanol, methanol, ethyl acetate and acids (Eudragit^®^ E100, Evonik Röhm GmbH & Co. KG, Darmstadt, Germany); sodium carboxymethylcellulose was a water soluble cellulose derivative (NaCMC, Fluka Biochemika, Medium viscosity, Sigma-Aldrich Chemie GmbH, Buchs, Switzerland), acetic acid glacial (Rochelle Chemicals, Johannesburg, South Africa) and deionized water.

### 3.2. Equipment

A Haake MARS (Modular Advanced Rheometer System) rheometer (Thermo Electron Corporation, Karlsruhe, Germany) fitted with a cone and plate geometry with a diameter of 35 mm and cone angle of 1° (sensor C35/1°) Ti was employed. The measurements were undertaken in controlled stress mode in oscillation. Data were acquired with RheoWin PC Software, version 3.

### 3.3. Synthesis of the Interpolyelectrolyte Complex

The methacrylate copolymer was milled and 0.84 g dissolved in 50 mL of 0.1, 0.4 and 0.8 N acetic acid while solutions of 1.68 g NaCMC were prepared by dissolving in 50 mL deionized water. A transparent methacrylate copolymer solution was added into a transperant NaCMC solution and stirred under vigorous agitation at ambient temperature. Samples were withdrawn at intervals during synthesis for analyses of rheological properties at the following sampling points:

*Sampling point I*: 30 s of stirring after methacrylate copolymer was added to NaCMC solution.

Sampling point II: 1 h later.

*Sampling point III*: At breaking point. Breaking point is regarded at the point during synthesis where the IPEC thickens in such a manner that magnetic stirring became a challenge. The interaction and blending is still non-homogenous but synthesis is on the verge of completion.

*Sampling point IV*: End of synthesis.

### 3.4. Rheological Studies

The IPEC at various sampling points was loaded onto the rheometer plate using a spatula. All tests were undertaken at the different sampling points to monitor the rheological changes and how these changes relate to the interactions such as intermolecular and intramolecular bondings taking place at each sampling point. Other than temperature ramp, all other studies were undertaken at 25 °C.

#### 3.4.1. Basic Viscosity

To determine the degree of resistance to flow at each interval of sampling as stated in Section 3.3; the samples were subjected to shear rate range of 0.00–200.00 s^−1^ over 180 s.

#### 3.4.2. Yield Stress Test

Yield stress is undertaken to obtain structural strength (yield value) of a system at rest beyond which it starts to flow. It was undertaken at the different sampling points to monitor the changes in yield stress from start to end of synthesis. It was also important to obtain the yield value for each sampling as it was employed in some of the other tests such as temperature ramp, frequency sweep; and creep and recovery test. Hence the yield stress test was undertaken within the shear stress range of 0.200–200 Pa over 200 s.

#### 3.4.3. Oscillatory Stress Sweep

One of the factors that influence the uptake of solvent and subsequent drug release from a polymeric matrix is the visco-elastic characteristics of the polymer utilized. Hence, oscillatory stress sweep was conducted over a shear stress of 0.100–70.00 Pa at frequencies 0.1, 1 and 10 Hz to determine the visco-elastic region of IPEC.

#### 3.4.4. Oscillatory Frequency Sweep

Oscillatory frequency sweep was utilized to determine the degree of elasticity and subsequent structural behavior of IPEC. The condition and consistency of the IPEC at each sampling point were assessed by undertaking frequency sweep from 50.00 to 0.05 Hz (314.2–0.3142 rad/s). The shear stress for each sample is dependent on the yield value obtained from yield stress test.

#### 3.4.5. Creep and Recovery Test

To assess the degree of deformation of IPEC on application of stress and its ability to recover, creep and recovery test was employed. It is used to monitor the resultant strain on a material after stress is applied. Stress was applied for 60 s and allowed to recover over 180 s.

#### 3.4.6. Temperature Ramp

The impact of temperature on IPEC structure during and after synthesis was monitored over a temperature range of 15–70 °C. A solvent trap was used to cover the cone and plate to prevent evaporation and drying of samples during the tests. The frequency employed was the frequency at which the yield value was obtained.

#### 3.4.7. Static Lattice Atomistic Simulations (SLAS)

All modeling procedures and calculations were performed using the HyperChem™ 8.0.8 Molecular Modeling System (Hypercube Inc., Gainesville, Florida, USA) and ChemBio3D Ultra 11.0 (CambridgeSoft Corporation, Cambridge, UK) at the 4 sampling points—IPEC-I, IPEC-II, IPEC-III, IPEC-IV. SLAS was undertaken to provide insight into the interactions between methacrylate copolymer and NaCMC, mechanisms involved in the formation of IPEC as well as their energy properties. The methacrylate copolymer (E100) was drawn using ChemBio3D Ultra in its syndiotactic stereochemistry as a 3D model whereas the structure of CMC (4 saccharide units) was built from standard bond lengths and angles using sugar builder module on HyperChem™ 8.0.8. Structure of acetic acid was built up with natural bond angles as defined in the HyperChem™ software. The models were energy-minimized using a progressive-convergence-strategy where initially the MM+ (Molecular Mechanics plus) Force Field was used followed by energy-minimization using the Amber 3 (Assisted Model Building and Energy Refinements) Force Field. The conformer having the lowest energy was used to create the polymer-polymer and polymer-solvent complexes. A complex of one polymer molecule with another was assembled by disposing the molecules in a parallel way, and the same procedure of energy-minimization was repeated to generate the final models: CMC, E100 and CMC-E100. Full geometry optimization was carried out in vacuum employing the Polak–Ribiere conjugate gradient algorithm until an RMS gradient of 0.001 kcal/mol was reached. For Molecular Mechanics calculations in vacuum, the force fields were utilized with a distance-dependent dielectric constant scaled by a factor of 1. The 1–4 scale factors used were electrostatic—0.5 and van der Waals—0.5 [41]. To generate the final models in solvated system the MM simulations were performed for cubic periodic boxes with the polymer-polymer complex (IPEC) at the center of the cubic box and the remaining free space filled with water and acetic acid (AcA). The same procedure of energy-minimization was repeated to generate the solvated models except that the force fields were utilized with a distance-independent dielectric constant with no scaling. For simplification, IPEC, IPEC-AcA_2_, IPEC-AcA_4_, and IPEC-AcA_8_ represented IPEC, IPEC in 0.2 N acetic acid, IPEC in 0.4 N acetic acid, and IPEC in 0.8 N acetic acid, respectively. In addition, the force field options in the AMBER were extended to incorporate cutoffs to Inner and Outer options with the nearest-image periodic boundary conditions and the outer and inner cutoffs were to ensure that there were no discontinuities in the potential surface [42].

#### 3.4.8. Drug Release Studies

Preliminary studies to assess the applicability of IPEC for controlled release were undertaken. IPEC was frozen for 24 h at −70 °C and lyophilized for 48 h. It was then milled with the model drug levodopa (100 mg) and incorporated into 500 mg of IPEC and directly compressed. Levodopa was selected as the model drug based on its inherent poor pharmaceutical formulation stability characteristics. Swelling was performed using gravimetric methods. Briefly, the tablet matrices were weighed, placed in pre-weighed wire baskets, submerged in 100 mL of 0.1 N HCl and placed in a shaker bath (Orbital Shaker incubator, LM-530, Laboratory & scientific equipment Co., South Africa) at 37 °C. Increase in weight was determined at the 24th hour. IPEC’s degree of swelling was compared to that of NaCMC. Drug release profiles for IPEC (at different normalities of acetic acid) and NaCMC were obtained using USP apparatus II dissolution system (Erweka DT 700, Erweka GmbH, Heusenstamm, Germany). The system was set at 37 °C and 50 rpm for temperature and stirring rate respectively using pH 1.5 as the medium (900 mL). Samples were withdrawn at pre-determined intervals and replaced with fresh medium each time to maintain sink conditions. The quantity of levodopa released was quantified using UV spectrophotometer (LAMBDA 25 UV/Vis spectrophotometer, PerkinElmer, Waltham, Massachusetts, USA). The studies were undertaken in triplicates.

## 4. Conclusions

The rheological investigations have been employed to monitor the visco-elastic and mechanical changes that occurred during synthesis of the IPEC. The polymer-polymer crosslinking of methacrylate copolymer and NaCMC improved their rheological properties. This was proven by monitoring the different stages of synthesis, which showed increased elastic modulus suggesting that the IPEC exhibited a solid-like behavior while NaCMC behaved like a viscous liquid. The tests undertaken in oscillation stress mode showed that a three-dimensional network was obtained from the complexation of a low viscous solution (methacrylate copolymer) and an entangled solution (NaCMC). The complex high yield and elastic behavior stipulates that IPEC formed will behave more like a solid with hard consistency and so may be employed in formation of matrices and possibly hydrogel for controlled release and not be used in spreadable pharmaceutical dosage forms such as creams. The *in silico* findings perfectly corroborated with the rheometrical data and subsequently the ability to achieve controlled zero-order release of levodopa from the IPEC matrix. These experimental findings also elucidated the interactions between NaCMC and methacrylate copolymer (E100) at the various sampling points of synthesis and demonstrated their influence on the rheological transitions.
